# Brain complexity in stroke recovery after bihemispheric transcranial direct current stimulation in mice

**DOI:** 10.1093/braincomms/fcae137

**Published:** 2024-05-13

**Authors:** Francesca Miraglia, Chiara Pappalettera, Saviana Antonella Barbati, Maria Vittoria Podda, Claudio Grassi, Paolo Maria Rossini, Fabrizio Vecchio

**Affiliations:** Brain Connectivity Laboratory, Department of Neuroscience and Neurorehabilitation, IRCCS San Raffaele, 00163, Rome, Italy; Department of Theoretical and Applied Sciences, eCampus University, Novedrate, 22060, Como, Italy; Brain Connectivity Laboratory, Department of Neuroscience and Neurorehabilitation, IRCCS San Raffaele, 00163, Rome, Italy; Department of Theoretical and Applied Sciences, eCampus University, Novedrate, 22060, Como, Italy; Department of Neuroscience, Università Cattolica del Sacro Cuore, 00168 Rome, Italy; Fondazione Policlinico Universitario A. Gemelli IRCCS, 00168 Rome, Italy; Department of Neuroscience, Università Cattolica del Sacro Cuore, 00168 Rome, Italy; Fondazione Policlinico Universitario A. Gemelli IRCCS, 00168 Rome, Italy; Department of Neuroscience, Università Cattolica del Sacro Cuore, 00168 Rome, Italy; Fondazione Policlinico Universitario A. Gemelli IRCCS, 00168 Rome, Italy; Brain Connectivity Laboratory, Department of Neuroscience and Neurorehabilitation, IRCCS San Raffaele, 00163, Rome, Italy; Brain Connectivity Laboratory, Department of Neuroscience and Neurorehabilitation, IRCCS San Raffaele, 00163, Rome, Italy; Department of Theoretical and Applied Sciences, eCampus University, Novedrate, 22060, Como, Italy

**Keywords:** stroke, entropy, tDCS, local field potential

## Abstract

Stroke is one of the leading causes of disability worldwide. There are many different rehabilitation approaches aimed at improving clinical outcomes for stroke survivors. One of the latest therapeutic techniques is the non-invasive brain stimulation. Among non-invasive brain stimulation, transcranial direct current stimulation has shown promising results in enhancing motor and cognitive recovery both in animal models of stroke and stroke survivors. In this framework, one of the most innovative methods is the bihemispheric transcranial direct current stimulation that simultaneously increases excitability in one hemisphere and decreases excitability in the contralateral one. As bihemispheric transcranial direct current stimulation can create a more balanced modulation of brain activity, this approach may be particularly useful in counteracting imbalanced brain activity, such as in stroke. Given these premises, the aim of the current study has been to explore the recovery after stroke in mice that underwent a bihemispheric transcranial direct current stimulation treatment, by recording their electric brain activity with local field potential and by measuring behavioural outcomes of Grip Strength test. An innovative parameter that explores the complexity of signals, namely the *Entropy*, recently adopted to describe brain activity in physiopathological states, was evaluated to analyse local field potential data. Results showed that stroke mice had higher values of Entropy compared to healthy mice, indicating an increase in brain complexity and signal disorder due to the stroke. Additionally, the bihemispheric transcranial direct current stimulation reduced Entropy in both healthy and stroke mice compared to sham stimulated mice, with a greater effect in stroke mice. Moreover, correlation analysis showed a negative correlation between Entropy and Grip Strength values, indicating that higher Entropy values resulted in lower Grip Strength engagement. Concluding, the current evidence suggests that the Entropy index of brain complexity characterizes stroke pathology and recovery. Together with this, bihemispheric transcranial direct current stimulation can modulate brain rhythms in animal models of stroke, providing potentially new avenues for rehabilitation in humans.

## Teaser

Miraglia *et al*. demonstrated—in a stroke mouse model—that brain activity changes, measured by Entropy index, correlate with stroke. They also demonstrated that transcranial direct current stimulation ameliorates post-stroke deficits and Entropy correlates with functional recovery. The authors concluded that Entropy could characterize stroke pathology and predict stroke outcomes.

## Graphical Abstract

**Graphical Abstract:**
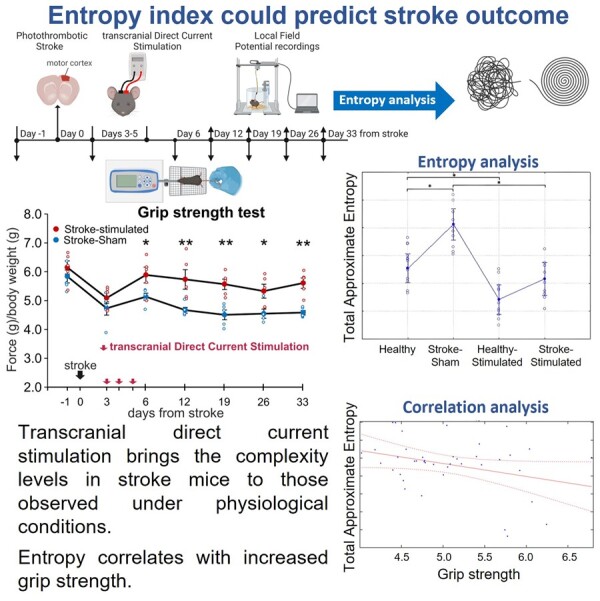

## Introduction

Stroke is one of the leading causes of motor disability.^1^ It occurs when the blood supplying the brain is suddenly disrupted reducing the supply of oxygen and glucose, resulting in brain tissue damage.^2^ This damage can lead to a wide range of physical and cognitive impairments, such as difficulty with movement, speech and memory.^3^ There are many different rehabilitation approaches aimed at improving clinical outcomes for stroke survivors. These may include physical therapy, occupational therapy, speech therapy, cognitive rehabilitation and non-invasive brain stimulation (NIBS).^4^ However, there is still no consensus on which approach is the most effective among others.

One reason for this lack of consensus is that stroke can affect individuals differently, depending on the location and extent of the brain damage.^2^ Additionally, stroke rehabilitation may involve a complex interplay of factors, including the timing and intensity of therapy, the type of treatment used and the individual’s overall health and well-being.^5^ As regards timing, an early *versus* a chronic intervention could improve recovery by boosting spontaneous plastic and neuroreparative mechanisms, occurring in the early post-stroke phase. However, physical therapy in the acute phase is often not practicable, especially when it comes to severe impairments. Conversely, the use of NIBS for early treatment appears more feasible. Among NIBS treatments, transcranial direct current stimulation (tDCS) has shown promising results to enhance motor and cognitive recovery in stroke survivals.^6-9^ This technique involves the application of a low-intensity electric current on the scalp passing the brain layers modulating in the end the neuronal activity inside the brain.

In the field of NIBS, it has been demonstrated that tDCS can modulate apoptosis, neuroinflammation and oxidative stress pathways.^10-12^ Several lines of evidence have also highlighted the effect of tDCS, especially anodal stimulation, on plasticity mechanisms both in human and animal models.^8,13-19^ In animal model of stroke, it has been also shown that both anodal and cathodal tDCS stimulate neurogenesis and reduce microglia activation and polarization towards the neurotoxic M1-phenotype while stabilizing microglia polarization towards the neuroprotective M2-phenotype.^20^ A reduced microglia activation in the perilesional region has also been proven following a very early application (i.e. 6 h after stroke) of cathodal tDCS in a mouse model of motor cortex stroke.^21^

Most of the studies focused on the effects of tDCS, which typically involves the application of the stimulation by a single hemispheric electrode; however, one of the most innovative methods of application is bilateral tDCS, also known as bihemispheric tDCS. It is a type of tDCS that involves the simultaneous application of anodal tDCS to one hemisphere of the brain and cathodal tDCS to the contralateral one.^22,23^ One potential advantage of bihemispheric tDCS is that it may provide more targeted and effective stimulation to the brain.^24^ By simultaneously increasing excitability in one hemisphere and decreasing excitability in the other hemisphere, bihemispheric tDCS creates a more balanced modulation of brain activity.^25,26^ This may be particularly useful in cases where there is an imbalance in brain activity, such as in stroke, in which the mutual transcallosal modulation is deranged with a decrease in excitability of the stroke hemisphere and an increase of the unaffected one.^27-29^

Currently, there are very few studies investigating the effects of bihemispheric tDCS in animal models of stroke through the analysis of brain electrical activity. Hence, there is a growing need to understand how bihemispheric stimulation modulates brain rhythms in animal models possibly providing new avenues for rehabilitation in stroke patients. Importantly, establishing a correlation between the efficacy of tDCS treatment on stroke recovery and the relative changes in EEG brain activity would corroborate the utility of electrical recordings to find novel biomarkers for stroke outcome.

Animal models are an essential tool in the stroke study because they enable researchers to investigate not only behavioural outcomes but also related tissue-level changes.^30^ In fact, they allow *in vivo* investigation of electrical activity in cerebral cortex providing a precious source for the identification of important biomarkers modulating the synaptic response to different therapies,^31^ and treatments.^32^

One of the most interesting and innovative approach to study tDCS modulatory effects on electrical brain activity is to analyse the measures of complexity as they are considered important to describe the condition of the brain. Entropy is defined as the level of information, surprise, uncertainty, or a gradual decline into disorder in a given amount of a signal.^33^ It was recently adopted with promising results to describe brain activity in physiological and pathological states and after rehabilitation treatments, demonstrating as it can be a useful parameter to describe the complexity of the brain.^34-36^

It provides valuable insights into the healthy aging brain's functionality, serving as a potential tool for identifying markers of healthy brain aging.^37,38^ Moreover, previous studies showed that Entropy measures offered valuable information about the changes in patients with Alzheimer’s disease and Mild Cognitive Impairment.^39,40^ The less complex EEG rhythms observed in these patients can be considered a neuro-marker, providing insight into the underlying mechanisms of cognitive decline.^38^ Furthermore, there is emerging evidence supporting the potential of Entropy analysis in EEG as a valuable tool for detecting early-stage Parkinson's disease and predicting cognitive decline in individuals affected by this disease.^41,42^ Additionally, previous investigations involving human subjects have consistently indicated heightened brain complexity in stroke patients in comparison to control subjects, during resting-state EEG recordings.^43,44^

Brain is a dynamic and complex system, therefore, the aforementioned methodology allows us to quantify and characterize the degree of disorder present in the system, allowing a better comprehension and representation of brain network dynamics.^34,35,37^ In comparison with other methods of analysis, several researchers have highlighted the numerous advantages of applying Entropy analysis to EEG signal dynamics.^45-48^ The heightened sensitivity of Entropy analysis to temporal dynamics allows for the discernment of changes in brain activity over time. Unlike conventional techniques such as power spectrum measures, which primarily focus on dissecting frequency components, or connectivity measures that assess interactions between brain regions, Entropy analysis offers a valuable advantage in understanding the complexities associated with brain activity. Moreover, Entropy measures intentionally embrace nonlinear complexity, distinguishing them from analyses that predominantly address linear relationships in brain signals. This nonlinear perspective is particularly pertinent for studying changes in brain dynamics, aiming for a more precise representation of alterations in EEG signals. Another notable feature is the robustness of Entropy measures across various frequency bands, enabling a holistic assessment of changes in brain activity across the entire frequency spectrum. Consequently, Entropy analysis provides valuable insights beyond the limitations of traditional techniques, revealing information that might go unnoticed with other methods.

Given these premises, the aim of the current study has been to evaluate brain complexity modulations in healthy and stroke mice subjected to bihemispheric tDCS treatment by combining results obtained from the analysis of electric brain activity through local field potential (LFP) recordings and the behavioural outcomes obtained in the grip strength test. Importantly, for the first time, we have analyzed the LFP data through an innovative parameter that explores the complexity of a signal, namely *Entropy*.

## Materials and methods

### Animals

A total of 42 C57BL/6 male mice (5–6 weeks old) were used. Experiments and animal procedures were authorized by the Catholic University Ethics Committee and were in line with Italian (Ministry of Health guidelines, Legislative Decree No. 116/1992) and European Union (Directive No. 86/609/EEC) legislations on animal procedures. Mice were randomly assigned to one of the following groups: mice subjected to stroke and tDCS (Stroke-tDCS) or sham stimulation (Stroke-Sham); mice that were not subjected to stroke and received tDCS (Healthy-tDCS) or sham stimulation (Healthy-Sham). Randomization was performed using random numbers. Groups were adjusted for age, weight, littermate conditions and baseline performances in the motor test.

All mice subjected to stroke met the inclusion criteria (i.e. significant impairments, defined as <93% of baseline performance in the grip strength test). No animal was excluded because of premature death related to technical complications.

### Photothrombotic stroke

Motor cortex infarct was performed by using the Rose-Bengal photothrombosis method as previously described.^25,49^ Briefly, mice were anesthetized with a cocktail of ketamine (87.5 mg/kg) and xylazine (12.5 mg/kg) and were placed in a stereotaxic frame. The skull surface was exposed and covered using a custom mask; only a circular area (3 mm^2^) over the left primary motor cortex remained exposed. Five minutes after the Rose-Bengal dye injection (75 µg/g of body weight, intraperitoneally injected) the exposed area was illuminated for 15 min by using a fibre optic bundle connected to a cold light source. After photothrombotic stroke induction, two epicranial plastic tubes (internal diameter 3 mm) were implanted over the motor cortex of both hemispheres for tDCS delivery as previously described.^25^ After surgery, animals were placed in individual cages where moist food was provided to facilitate food intake.

### Transcranial direct current stimulation

During the sub-acute phase of ischaemic stroke (i.e. 72 h after stroke) mice were subjected to bihemisperic tDCS, with the anode over the lesioned motor cortex (left hemisphere) and the cathode over the contralateral side.^25^ Epicranial cannulae were filled with saline solution (NaCl 0.9%) and connected to a battery-driven apparatus (BrainSTIM, EMS, Italy) delivering constant current. tDCS was applied for 20 min per day for three consecutive days at a current intensity of 250 μA (current density of 35.4 A/m^2^). The current intensity was ramped for 10 s to avoid a stimulation break effect. Electrode montage and current density were like those recently adopted for rodent models and close to the recommended safety limits in rodents.^50^ No abnormal behaviour was observed related to the stimulation. Sham-stimulated animals underwent the same manipulations as in the ‘real’ stimulation condition, but no current was delivered.

### LFP recordings

Mice were anesthetized by an intraperitoneal injection of ketamine (87.5 mg/kg) and xylazine (12.5 mg/kg) cocktail and located in a stereotaxic frame. Twenty-four hours after the end of stimulation protocol the epicranial cannulae were removed and recording electrodes were implanted. Briefly, four small drawl holes were made at stereotaxic coordinates corresponding to primary motor cortex (+0.5 mm antero-posterior and 1.75 mm lateral to the bregma) and somatosensory cortex (+0.4 mm antero-posterior and 2.5 mm lateral to the bregma) of both sides. Other two holes were drilled: one for the reference electrode that was laid at the centre of the parietal bone and one over the cerebellum to ease the insertion of a skull screw used as ground. Six stainless steel filaments connected to a multipin socket (NPD-18-DD-GS connector, Omnetics), were then laid through the burr holes over the primary motor and somatosensory cortices to obtain an electrical contact without lesioning the dura mater, avoiding brain trauma and the risk of a cerebrospinal fluid leak. The whole implant was finally ensured with dental light-curing resin (Tetric Evoflow®). Mice were allowed to recover for 6 days during which they were monitored for any sign of pain or distress. At the end of this period, the animals were individually placed in a recording cage and allowed to freely move while LFPs were recorded. Data acquisition was carried out at 1, 2, 3 and 4 weeks after the end of tDCS or sham stimulation protocol by using the Cereplex Direct system (Blackrock microsystem). Each recording session lasted 30 min and each LFP was sampled at 500 Hz.

### LFP data preprocessing

The LFP data were preprocessed using a home-made MATLAB software, based on EEGLAB toolbox codes (Swartz Center for Computational Neurosciences, La Jolla, CA, USA). The LFP data were band-pass filtered with a finite impulse filter (FIR) applied to extract data in the frequency range from 0.2 to 47 Hz.^51,52^ LFP continuous data were segmented in 2 sec length epochs and trials with artefact activity or aberrant waveforms (i.e. movements and environmental artefacts) were removed by an expert data visual inspection. At the end of the artefact removal procedure, at least 23 min of recording remained for each mouse and condition.

### Entropy analysis

The complexity of brain activity was studied by entropy measures. Entropy, a widely applied measure in science and engineering, initially emerged in thermodynamics to elucidate a system's tendency to transition from higher energy (less probable) to lower energy (more probable) states.^53,54^ Subsequently adapted for signal analysis, Entropy serves as a metric quantifying the information within a signal by assessing the predictability of future values based on the observed probability distribution within the signal itself.^55,56^

Over recent decades, there has been a substantial growth of interest in Entropy analysis, with its application extending to diverse research fields, including neuroscience. The nonlinear chaotic behaviour inherent in neural systems justifies the application of methods from the theory of nonlinear dynamics to assess cerebral activity and quantify its variability.^56^ This variability arises from the intricate interplay between individual neurons and their neural networks, spanning extensive spatiotemporal scales in the brain. Moreover, significant findings link this variability to the brain's self-organized criticality, where information capacity is maximized.^34,35,57^

The increasing use of Entropy analysis reflects its growing importance in unravelling cerebral dynamics and holds promise for advancing our understanding across various domains.

In this study, we employed the measure of Approximate Entropy (ApEn), that has many advantages: it maintains good reproducibility when used with time series, it is almost unaffected by noise, and it detects changes in underlying episodic behaviour undetected by peak occurrences or amplitudes.^36,58,59^ ApEn values were computed, in each mouse, for each channel and for each epoch using a homemade MATLAB software. For each LFP recording, those values were averaged among epochs to obtain a single ApEn value for each channel.

The homemade software estimates ApEn dimensionless values. The higher the value of ApEn, the more irregular and less predictable the signal is. On the other hand, the lower this value, the more periodic and stable the signal tends to be. In the ApEn analysis two input parameters need to be defined: a model length (*m*) and a tolerance factor (*r*), also called similarity factor, used to identify a range of similarity similarities between data points. In this study, *m* and *r* were set equal to the default MATLAB values: thus, *m* = 2 and *r* = 0.2 * variance (*x*), were used, in which *×* corresponds to an epoch of length of 2 sec of a specific channel. The obtained ApEn values range from 0 (regular time series) to 2 (random time series).^60,61^

In particular, the calculation of ApEn is described as follows:^54,62,63^

1. A point-by-point comparison is made between each data sequence of length m and all other sequences. If the distance between points is less than the tolerance factor r a match is scored.

All the matches are counted as described by the expression (1):


(1) $$\;\;\;\;\;\;\;\;\;\;\;\;\;N_{i}=\sum_{i=1,\;\;i\neq k}^{N}(Y_{i}-Y_{k\infty }<r)$$

where $Y_{i}$ is the m-dimensional vector sequence, defined from as a delayed reconstruction of the time series {y(i)}=y(1),y(2),…,y(N), where i ranges from 1 to *N*, number of data points:


(2) $$\;\;\;\;\;\;\;\;\;\;\;Y_{i}=\;[y(i),\;y(i+1),\;\ldots ,\;y(i+m+1)]$$

2. The comparison is performed on each successive *m* + 1-long sequence, starting from the first sequence of m + 1 points, as shown in the Eq. (2).
3. The number of matches is converted to a natural logarithm value, and afterwards normalized by the number of data points (*N*):
  (3) $$\;\;\;\;\;\;\;\;\phi_{m}=\;(N\;-m+1{)}^{-\;1}\overset{N-m+1}{\underset{i=1}{\sum }}\;\log\;(N_{i})$$

Finally, the ApEn is calculated using the following expression:


(4) $$\;\;\;\;\;\;\;\;\;\;\;\;ApEn=\;\phi_{m}-\;\phi_{m\;+1}\;.\;$$

ApEn values were estimated for the 4 time points (at 1, 2, 3 and 4 weeks after the end of tDCS) in the whole brain (Total ApEn), averaging the values among the four available channels and among the two available channels for each hemisphere (Hem ApEn) to obtain the computation of entropy in the right and left hemisphere of the brain.

#### Motor function assessment

**Figure 1:**
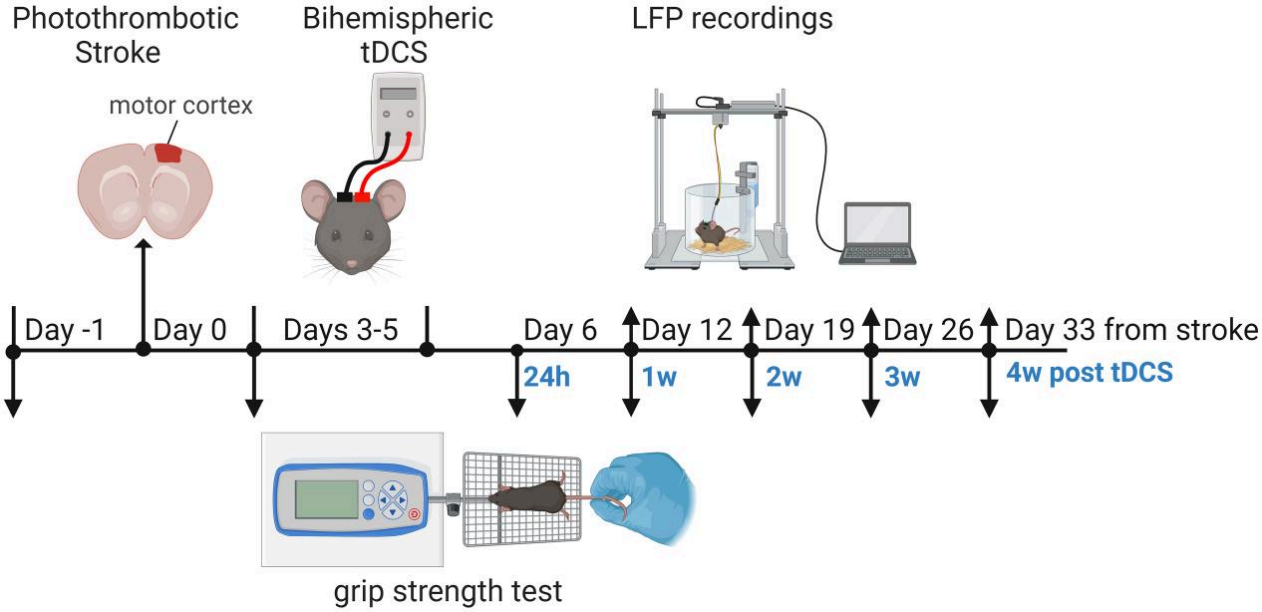
**Timeline of experimental protocols.** Timeline shows experimental design with respect to stroke induction (days from stroke) and to the end of tDCS or sham treatment (hours or week post tDCS). All mice enrolled in the study were subjected to LFP recordings; a subgroup of mice (*n* = 5 Stroke-tDCS; *n* = 5 Stroke-Sham) were also tested in the forelimb grip strength test. Figure was created with BioRender.com.

A grip strength metre (GSM, Bioseb Instrument) was used to assess forelimb grip strength. Animals were placed in the test room for about 30 min to let them familiarize the environment. Mice were held by the tail and allowed to grasp a wire grid with their forepaws. They were then pulled backwards by the tail until they released the grid. Grip strength was expressed as the mouse forelimb force measured in grams by the GSM, divided by grams of body weight.^17,25^ The average of three consecutive attempts was used for statistical analysis. Motor test was performed the day before stroke induction to assess baseline value, 72 h after stroke and before tDCS or sham stimulation to assess motor deficits and 24 h and at 1, 2, 3 and 4 weeks after stimulation (few hours before LFP recordings) to evaluate tDCS efficacy on motor recovery. Figure 1 shows the timeline of the entire experimental protocol.

#### Statistical analysis

The sample size was determined with the GPower 3.1.9.4 software considering the mean ± SD of two groups, based on our previous study on entropy measures in human physiological ageing^43^ and considering a power of 80%, a confidence interval of 95% and a type I error of 0.05 with two tails.

The analyses were conducted with a statistical cut-off level of *P* < 0.05. Analysis of Variance (ANOVA) was implemented with the software Statistica (StatSoft Inc.). The normality of the data was tested using the Kolmogorov-Smirnov test, and the hypothesis of Gaussianity could not be rejected. ANOVA was chosen since it is known to be robust for the departure of normality and homoscedasticity of data being treated. Greenhouse and Geisser correction was used for the protection against the violation of the sphericity assumption in the repeated measure ANOVA. Moreover, the post-hoc analysis was performed using Duncan’s or Bonferroni test and a 0.05 significance level. Experimental unit was a single animal; investigator/observer/outcome assessor was unaware of the group allocation.

The main aim of the study was to assess the possible changes in the Total ApEn after tDCS protocol (real and sham) in healthy and stroke mice. With this purpose, LFP recordings were performed in mice subjected to tDCS or sham stimulation at four time points (at 1, 2, 3 and 4 weeks) after the end of tDCS protocol (Stroke-tDCS, *n* = 9; Stroke-Sham, *n* = 10; Healthy-tDCS, *n* = 11; Healthy-Sham, *n* = 12). To do this, the ANOVA design among Groups (Healthy-Sham, Stroke-Sham, Healthy-tDCS, Stroke-tDCS) and Time Points (1, 2, 3 and 4 weeks after stimulation) was computed for the evaluation of the Total ApEn over the whole brain between the experimental groups.

The ANOVA among the factors Hemispheres (Affected, Unaffected), Groups (Healthy-Sham, Stroke-Sham, Healthy-tDCS, Stroke-tDCS) and Time Points (1, 2, 3 and 4 weeks after stimulation) was employed for the evaluation of the differences between the values of entropy in the two hemispheres (Hem ApEn).

To establish whether Total ApEn correlated with functional recovery after stroke, a subgroup of mice undergoing LFP recordings (*n* = 5 Stroke-tDCS mice; *n* = 5 Stroke-Sham mice) were also tested in the forelimb grip strength to assess their motor performance. Since we previously demonstrated that mice subjected to bihemispheric tDCS showed amelioration of post-stroke skilled and nonskilled motor performance as well as of forelimb strength,^25^ here we limited our functional analysis to the assessment of forelimb strength that could be measured by using a no stressful and easy test. Thus, a further two-way Repeated Measure (RM) ANOVA (repetition factor, time) followed by Bonferroni *post hoc* was used to analyze grip strength data.

Moreover, Pearson’s linear correlation analysis between the Total ApEn and grip strength, and between the Hem ApEn and the grip strength was computed for all the conditions (*n* = 5 Stroke-tDCS mice; *n* = 5 Stroke-Sham mice) and time points (at 1, 2, 3 and 4 weeks after stimulation).

## Results

**Figure 2:**
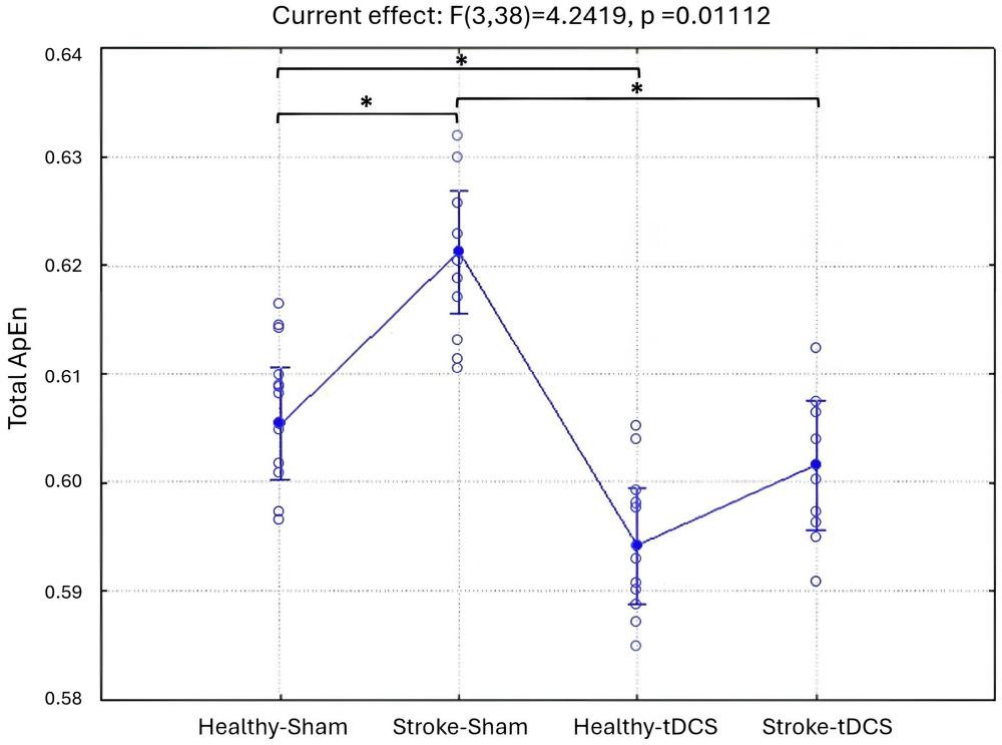
**Total ApEn results.** The ANOVA for the evaluation of Total ApEn between the experimental Groups and Time Points showed a statistically significant interaction (F(3,38) = 4.2419, *P* = 0.01112) for the factor Groups. Total Approximate Entropy (Total ApEn) values in Healthy-Sham (*n* = 12 mice), Stroke-Sham (*n* = 10 mice), Healthy-tDCS (*n* = 11 mice) and Stroke-tDCS (*n* = 9) groups are reported in the figure. Vertical bars represent the standard errors. *represents the significant results *P* < 0.05; ANOVA followed by Duncan *post hoc*.

The ANOVA for the evaluation of Total ApEn between the experimental Groups and Time Points showed a statistically significant interaction (F(3,38) = 4.2419, *P* = 0.01112) for the factor Groups (Fig. 2). In particular, the Duncan *post hoc* analysis revealed that the Stroke-Sham group (*n* = 10 mice) presented higher values of Total ApEn respect to Healthy-Sham (*n* = 11 mice; *P* = 0.05), as the stroke has the effect to increase brain complexity and signal disorder.

Furthermore, the Stroke-tDCS group (*n* = 9 mice) exhibited lower values of Total ApEn respect to the Stroke-Sham (*P* = 0.021) and this trend is visible also in the Healthy-tDCS respect to Healthy-Sham (*n* = 12 mice; *P* = 0.06), as the stimulation produced the same effect through the brain layers leading to reduced complexity in the signals regardless the pathology.

**Figure 3:**
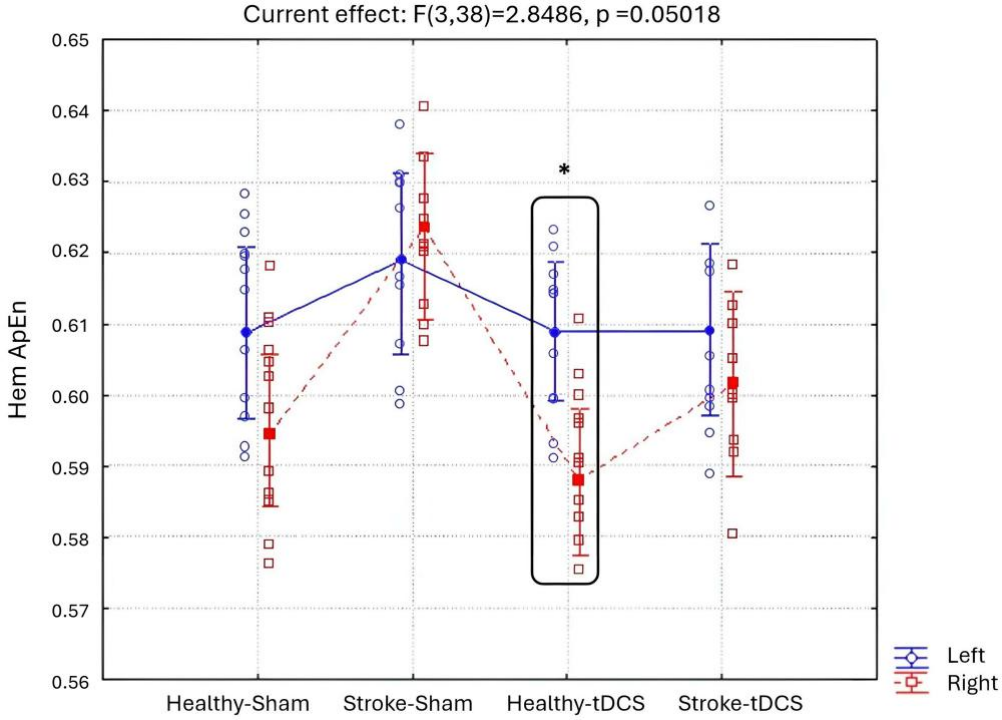
**Hem ApEn results.** The ANOVA for the evaluation of Total ApEn between hemispheres (Left Hem ApEn, Right Hem ApEn), Groups (Healthy-Sham, Stroke-Sham, Healthy-tDCS, Stroke-tDCS) and time points (1, 2, 3 and 4 weeks after stimulation) showed a significant interaction (F(3, 38) = 2.8486, *P* = 0.050) between Hemispheres and Groups. Total ApEn values computed in the left (Left Hem ApEn, red line) and in the right hemisphere (Right Hem ApEn, blue line) in Healthy-Sham (*n* = 12 mice), Stroke-Sham (*n* = 10 mice), Healthy-tDCS (*n* = 11 mice) and Stroke-tDCS (*n* = 9 mice) groups are reported in the figure. Vertical bars represent the standard errors. *represents the significant results *P* < 0.05; ANOVA followed by Duncan *post hoc*.

The ANOVA for the evaluation of Total ApEn between hemispheres (Left Hem ApEn, Right Hem ApEn, being left hemisphere the lesioned one in the Stroke groups), Groups (Healthy-Sham, Stroke-Sham, Healthy-tDCS, Stroke-tDCS) and time points (1, 2, 3 and 4 weeks after stimulation) showed a significant interaction (F(3, 38) = 2.8486, *P* = 0.050) between Hemispheres and Groups (Fig. 3). Interestingly, the Duncan *post hoc* analysis showed a decrease of right Hem ApEn values respect to left Hem ApEn one in Healthy-tDCS group (*P* = 0.005). Furthermore, it is worth mentioning that a similar trend of decreased right Hem ApEn values compared to left Hem ApEn was observed in Stroke-tDCS mice, although the statistical significance was only marginal (*P* = 0.3).

**Figure 4:**
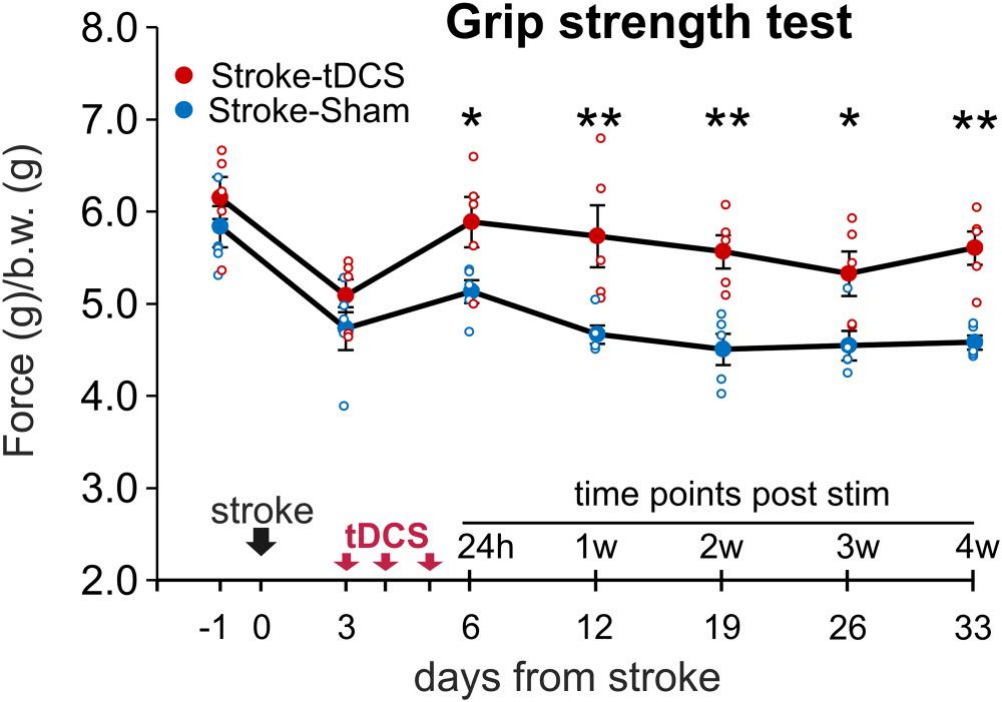
**Time course of post-stroke recovery in the grip strength test in mice subjected tDCS and sham stimulation**. Stroke-tDCS mice (*n* = 5) displayed higher forelimb strength values than Stroke-Sham mice (*n* = 5), starting from day 6 after stroke (24 h post stim) and throughout the entire follow-up period. Data are expressed as mean ± SEM. *P < 0.05; **P < 0.001; two-way RM ANOVA, followed by Bonferroni *post hoc*. b.w. indicates body weight.

Regarding the forelimb strength, as expected, 72 h after stroke it was significantly decreased in both tDCS and sham groups (*P* < 0.001 versus baseline; two-way RM ANOVA, Bonferroni *post hoc*; Fig. 4). Of note, in line with previously published data, mice subjected to tDCS displayed improved grip strength performance compared to Stroke-Sham mice starting from 24 h after the completion of the tDCS protocol, and lasting throughout the follow-up period (main factor stimulation: F (1, 48) = 15.1; *P* = 0.005; *P* < 0.05 Stroke-tDCS versus Stoke-Sham at all time points tested following stimulation; two-way RM ANOVA, Bonferroni *post hoc*, Fig. 4).

**Figure 5:**
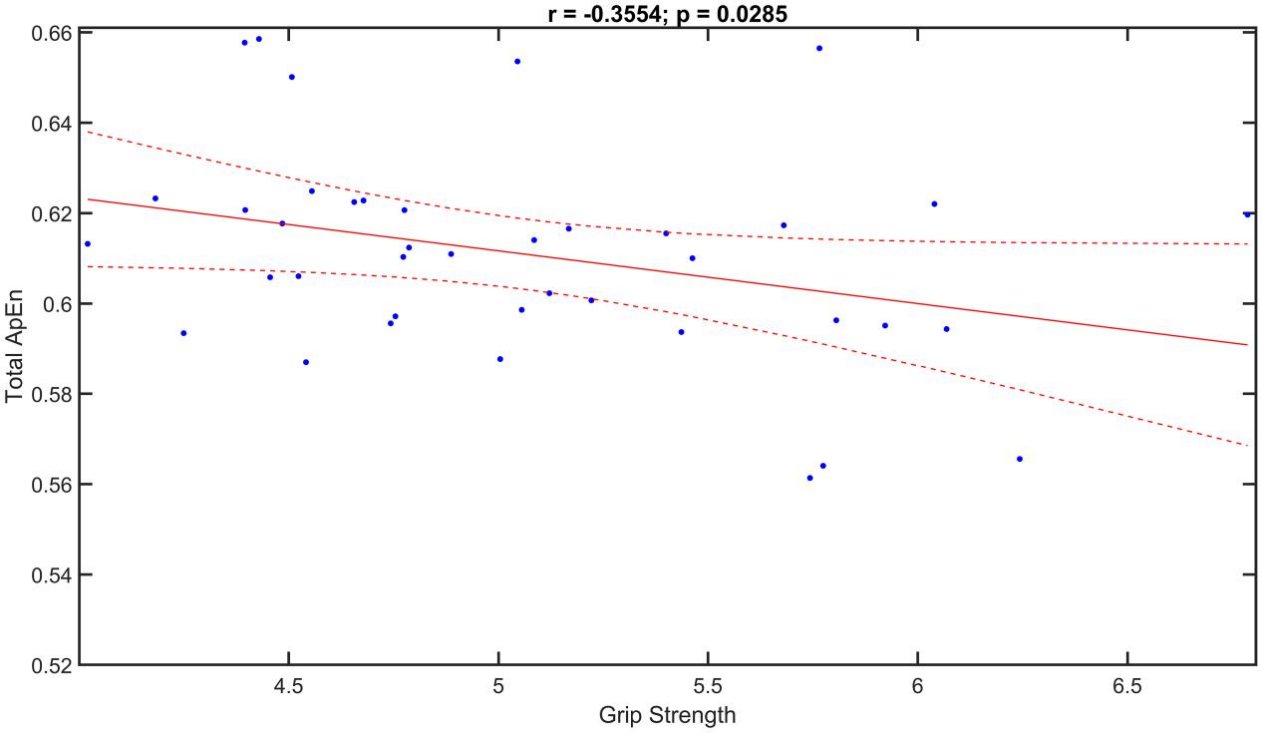
**Correlation analysis between total ApEn and grip strength.** Scatterplots of Pearson’s correlation analysis between Total ApEn and Grip Strength values (*r* = −0.3554, *P* = 0.0285) in all mice tested (*n* = 5 Stroke-tDCS mice; *n* = 5 Stroke-Sham mice) and time points (at 1, 2, 3 and 4 weeks after stimulation).

When we combined Total ApEn and Grip Strength, the results showed a negative correlation (*r* = −0.3554, *P* = 0.0285), namely higher the Total ApEn values, lower the Grip Strength engaged (Fig. 5).

**Figure 6:**
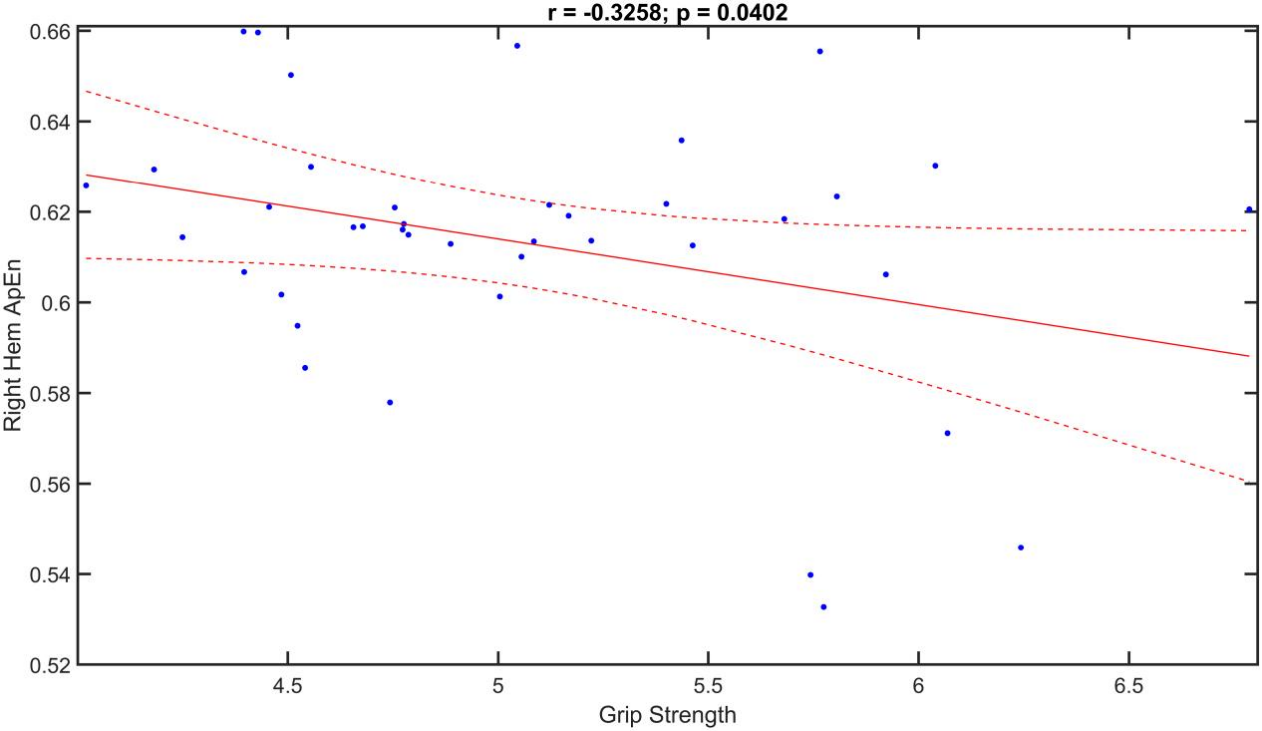
**Correlation analysis between hem ApEn and grip strength.** Scatterplots of Pearson’s correlation analysis between the right Hem ApEn and the Grip Strength values (*r* = −0.3258, *P* = 0.0402) in all mice tested (*n* = 5 Stroke-tDCS mice; *n* = 5 Stroke-Sham mice) and time points (at 1, 2, 3 and 4 weeks after stimulation).

Besides, the correlation analysis between the Hem ApEn and the Grip Strength revealed significant negative trend only between the right Hem (i.e. the hemisphere contralateral to the lesioned one) ApEn and Grip Strength (*r* = −0.3258, *P* = 0.0402), namely higher the right Hem ApEn values, lower the Grip Strength engaged (Fig. 6).

## Discussion

This study aimed to propose a novel methodological approach utilizing an innovative index, known as *Entropy*, to explore the complexity of electrical signals in the brain. *Entropy*, initially a concept in thermodynamics, recently found application in brain signal analysis, as a valuable tool for quantifying the randomness or disorder within the dynamic system of the brain. In particular, Carhart-Harris’ model further categorized brain states as ‘low Entropy’ or ‘high Entropy,’ where high Entropy implies increased disorder and lower energy demand.^35^ Applied to brain activity analysis, Entropy helps conceptualize the brain's neural population as a system operating in a baseline firing state, capable of transitioning to different firing states in response to stimuli, aging or pathological conditions. This transition may lead to a less complex system with a more regular firing pattern or a more complex one characterized by increased neural activity randomness.

Specifically, this research investigated the brain electrical activity modulations induced by bihemispheric tDCS in both healthy and stroke mice. The use of a mouse model of stroke has many advantages, allowing a strict control of possible confounding factors and a clearcut correlation between brain activities from selected areas and functional outcome measures. In particular, our experimental model allows: (i) to induce a reproducible focal ischaemia specifically targeting the forelimb motor cortex; (ii) to study a well characterized loss of function dependent on the selected brain area and, therefore, to better evaluate its recovery following treatment; (iii) to obtain accurate epidural EEG; (iv) to pave the way for future studies to identify cellular and molecular substrates of changes in brain activity following stroke and tDCS, that cannot be assessed in human subjects.

Brain represents a dynamic system that exhibits non-linearity, no stationarity and complexity so, the entropy-based measures are able to quantify the amount of disorder present in a system, enabling a more comprehensive characterization of the dynamics of brain networks.^48,64,65^ In fact, some hypotheses stated that the brain maintains a dynamic balance between order and randomness in its signalling. Too much order may lead to rigidity and reduced adaptability, while excessive randomness may result in chaotic, inefficient processing. Optimal cognitive function is often associated with a balanced level of signal complexity.^57,66^ Accordingly, assessing the degree of complexity can provide insights into the brain’s different conditions, reflecting its dynamic nature.^43,57,67^

The results of the present study revealed that the groups of stroke mice exhibited higher values of Total ApEn compared to the healthy groups. This finding suggests an increase in brain complexity and signal disorder resulting from the stroke. This outcome confirms our initial hypothesis, demonstrating that assessing brain complexity is a valuable tool for characterizing normal and stroke-related brain modulations. Notably, prior studies on human subjects have also reported an increased brain complexity in stroke patients compared to control subjects during resting-state EEG recordings.^68,69^ It was found that Sample Entropy in ischaemic thalamus of stroke subjects was higher at all electrodes with respect to control subjects while resting with eyes closed.^70,71^ The study by Hadiyoso and collaborators^43^ proposed a method for characterizing EEG signals in poststroke patients with cognitive impairment and normal subjects, by measuring EEG spectral power complexity. The key finding included a relationship between Spectral Entropy values and the severity of dementia, demonstrating the ability to differentiate between normal subjects and poststroke patients with cognitive impairment, suggesting its potential as a discriminative tool in this context. A further study aimed to assess the effectiveness of EEG-related indexes in distinguishing stroke patients from control participants and explore pathological EEG changes following chronic stroke. The fuzzy ApEn demonstrated high classification accuracy (84.85%), sensitivity (85.00%), and specificity (84.62%), suggesting the potential of EEG complexity as a robust discriminator for identifying stroke patients.^69^

Additionally, the results of the current study showed that after tDCS the complexity of the LFP signal, expressed by the Total ApEn index, was reduced in both healthy and stroke mice. Our results are in line with previous study,^72^ in which it was demonstrated that stroke-induced alterations in signal complexity, as evidenced by multiscale Entropy measures. Interestingly, the study showed that after anodal tDCS, there was a decrease in multiscale Entropy in the slow activity and an improvement in accuracy in a performed task, suggesting that these changes indicated a reversal of the pathological abnormalities observed after stroke. The authors further supported the notion that tDCS has the potential to impact and ameliorate brain activity observed in stroke-related conditions.

In a further study, the impact of anodal tDCS over the left lip region of the primary motor cortex on subacute post-stroke patients with apraxia of speech was investigated.^73^ Patients were randomized into either the tDCS group or the control group (received sham tDCS), both coupled with speech and language therapy. Using EEG assessments, cortical interconnections were measured using the non-linear index of cross ApEn. Following treatment, EEG analysis revealed modification in the cross ApEn (thus stronger connections) exclusively in the left hemisphere for the tDCS group, specifically in the of the speech articulation network, demonstrating a significant restore of speech functionality. All these studies support the translational value of our experimental model.

Interestingly, our analysis comparing the two hemispheres revealed that tDCS led to a decrease in ApEn values specifically in the right hemisphere compared to the left one, in both healthy and stroke mice. Notably, as the right hemisphere was stimulated with cathodal tDCS, these findings suggest that, in the context of bihemispheric tDCS, the cathodal stimulation played a more prominent role in reducing brain complexity. This effect was more pronounced in healthy mice compared to stroke mice. Although both groups exhibited a reduction in brain complexity with cathodal stimulation, a milder effect was seen in stroke mice compared to the healthy mice. This disparity in response could be attributed to the compromised brain activity and interhemispheric imbalance caused by the stroke, potentially delaying the full impact of the stimulation.

In a previous study using the same animal model of stroke and bihemispheric tDCS, we demonstrated by LFP recordings and connectivity analysis that functional coupling between primary motor and somatosensory cortices of both hemispheres was decreased at all frequency bands in stroke compared to healthy mice and, more importantly, tDCS significantly increased connectivity.^25^ Particularly, in stroke mice tDCS restored connectivity to values similar to those observed in healthy mice, suggesting that a structural network reorganization occurs following tDCS. At cellular and molecular levels, it was found that in the peri-infarct area (under anodal stimulation) tDCS enhanced BDNF expression and BDNF-dependent signalling pathways activation, resulting in increased structural plasticity, which might be the substrate of increased connectivity.

Here, based on ApEn analysis, we extended previous data highlighting a relevant role of cathodal stimulation applied over the contralateral hemisphere in reducing signal Entropy. It could be speculated that such stimulation likely rebalanced interhemispheric mutual modulation via transcallosal connections, which might support rescued excitability and plasticity mechanisms. Further studies are needed to characterize changes at cellular/molecular levels occurring in the motor cortex under cathodal stimulation.

Moreover, our study corroborates previous findings demonstrating that bilateral stimulation with simultaneous facilitating and inhibiting currents applied to the affected and unaffected motor areas, respectively, leads to a beneficial rebalance of interhemispheric activity, resulting in functional improvements.^74,75^ This knowledge contributes to a deeper understanding of the mechanisms underlying the therapeutic potential of tDCS and emphasizes the importance of considering network-level effects when designing and implementing tDCS interventions.

Indeed, in the current study motor function assessment revealed an interesting pattern in grip strength among the tDCS and Sham groups of stroke mice, which correlates with ApEn. Initially, as expected, there were no significant differences in grip strength between the two groups before and 72 h post stroke. However, 24 h post-stimulation, a significant difference emerged, showing better performance of mice subjected to tDCS, which persisted for an extended period of four weeks. These findings raise several speculations about the long-term effects of tDCS on stroke recovery. One possible interpretation is that tDCS may have an impact on neuroplasticity. The fact that grip strength differences became visible after 24 h, in line with what previously shown by Longo *et al.,*^25^ suggests that tDCS might trigger cascading physiological changes that gradually enhance motor function over time. The likelihood to engage such mechanisms is certainly enhanced by early application of tDCS. In this critical window, tDCS treatment has the potential to recruit, to some extent, the original functional network, contrasting the establishment of maladaptive plasticity mechanisms, which are known to hinder therapy-driven motor recovery in the chronic stage.

Previously, it was well-documented in the literature that photothrombotic cortical lesions result in grip strength deficits.^76^ Notably, our findings demonstrate that tDCS has the potential to mitigate the grip strength deficits associated with stroke.

Interestingly, our correlation analysis revealed a negative relationship between Total ApEn and grip strength, indicating that lower Total ApEn values were associated with higher grip strength engagement. More importantly, negative correlation was found also between right Hem ApEn and grip strength, indicating that lower right Hem ApEn values were related to higher grip strength.

The latter result could suggest a major role of cathodal stimulation in promoting recovery, as tDCS improves performance and reduces Entropy in the right hemisphere, which is undergoing cathodal stimulation. It could be hypothesized that cathodal stimulation down-regulates the over-activity in the right hemisphere, leading to a reduction in Entropy (increased order) and potentially promoting the improvement in grip strength.

In a more general view, this observation suggests that Entropy could serve as a biomarker of functional state following stroke. It opens new opportunities for research, focusing on exploring the intricate relationship between brain Entropy and motor recovery in stroke. Additionally, exploring the relationship between neural Entropy and motor function could provide valuable insights into the biomarkers of stroke recovery and potentially guide the development of targeted therapies and rehabilitative treatments.

Notwithstanding, the unilateral lesioning (specifically, the left side) introduces a consideration regarding hemisphere dominance and functions. While unilateral lesions offer a controlled and targeted approach to investigate the consequences of damage on specific functions associated with the left hemisphere, we recognize that this design may introduce a limitation into the generalization of these results. We acknowledge the importance of considering homogenous cohorts in future studies, emphasizing the need for balanced representation on both sides of the lesion.

## Conclusions

The current study stands out as the first randomized sham-controlled trial that applies bihemispheric tDCS over the motor cortex of mice, with an evaluation of LFP modulations over time using a novel index of brain complexity. Overall, this study contributes to the growing body of knowledge in the field of stroke rehabilitation and highlights the potential benefits of bihemispheric tDCS. By employing animal models and novel analysis techniques, we aim paving the way for further advancements in stroke rehabilitation strategies to improve the outcomes and quality of life for individuals affected by stroke.

## Data Availability

The data that support the findings of this study are available from the corresponding author on request.
